# New Frontiers in Diagnosis and Therapy of Circulating Tumor Markers in Cerebrospinal Fluid In Vitro and In Vivo

**DOI:** 10.3390/cells8101195

**Published:** 2019-10-02

**Authors:** Olga A. Sindeeva, Roman A. Verkhovskii, Mustafa Sarimollaoglu, Galina A. Afanaseva, Alexander S. Fedonnikov, Evgeny Yu. Osintsev, Elena N. Kurochkina, Dmitry A. Gorin, Sergey M. Deyev, Vladimir P. Zharov, Ekaterina I. Galanzha

**Affiliations:** 1 Laboratory of Biomedical Photoacoustics, Saratov State University, 83 Astrakhanskaya St, 410012 Saratov, Russia; mouse-oa@rambler.ru (O.A.S.); r.a.verhovskiy@mail.ru (R.A.V.); gafanaseva@yandex.ru (G.A.A.); fedonnikov@mail.ru (A.S.F.); dr_osintsev@mail.ru (E.Y.O.); e.katamadze@yandex.ru (E.N.K.); zharovvladimirp@uams.edu (V.P.Z.); 2 Arkansas Nanomedicine Center & Winthrop P. Rockefeller Cancer Institute, University of Arkansas for Medical Sciences, Little Rock, AR 72205, USA; msarimollaoglu@uams.edu; 3 Saratov State Medical University, 112 Bolshaya Kazachia St., 410012 Saratov, Russia; 4 Laboratory of Biophotonics, Skolkovo Institute of Science and Technology, 3 Nobelya Str., 121205 Moscow, Russia; gorinda@mail.ru; 5 Shemyakin-Ovchinnikov Institute of Bioorganic Chemistry, Russian Academy of Sciences, Miklukho-Maklaya St., 16/10, Moscow 117997, Russia; biomem@mail.ru; 6 Laboratory of Lymphatic Research, Diagnosis and Therapy (LDT), University of Arkansas for Medical Sciences, Little Rock, AR 72205, USA

**Keywords:** cerebrospinal liquid biopsy, in vivo flow cytometry, tumor biomarkers, circulating tumor cells, ctDNA, miRNA, exosomes, emboli, targeted therapy

## Abstract

One of the greatest challenges in neuro-oncology is diagnosis and therapy (theranostics) of leptomeningeal metastasis (LM), brain metastasis (BM) and brain tumors (BT), which are associated with poor prognosis in patients. Retrospective analyses suggest that cerebrospinal fluid (CSF) is one of the promising diagnostic targets because CSF passes through central nervous system, harvests tumor-related markers from brain tissue and, then, delivers them into peripheral parts of the human body where CSF can be sampled using minimally invasive and routine clinical procedure. However, limited sensitivity of the established clinical diagnostic cytology in vitro and MRI in vivo together with minimal therapeutic options do not provide patient care at early, potentially treatable, stages of LM, BM and BT. Novel technologies are in demand. This review outlines the advantages, limitations and clinical utility of emerging liquid biopsy in vitro and photoacoustic flow cytometry (PAFC) in vivo for assessment of CSF markers including circulating tumor cells (CTCs), circulating tumor DNA (ctDNA), microRNA (miRNA), proteins, exosomes and emboli. The integration of in vitro and in vivo methods, PAFC-guided theranostics of single CTCs and targeted drug delivery are discussed as future perspectives.

## 1. Introduction

Leptomeningeal and brain metastasis (LM and BM) as a result of metastatic dissemination of solid tumors (e.g., melanoma, breast cancer, lung cancer and colorectal cancer) and hematological neoplasms as well as primary brain tumors (BTs, e.g., glioma) are commonly fatal with minimum treatment options [1,2,3,4,5,6,7,8,9,10,11]. Relatively high number of underdiagnosed LM, BM and BT and often ineffective therapy are the major challenges. For example, autopsy data demonstrate that BM contribute to death in ~75% of melanoma patients but they are clinically diagnosed in only 37% cases [8].

Among other parts of central nervous system (CNS), cerebrospinal fluid (CSF) is the easiest accessible medium that can directly uptake tumor markers from different parts of CNS [12,13,14,15,16,17]. Normally, CSF is a colorless liquid (a total volume of 130–150 mL for human) that contains up to 5 cells/µL, mainly leukocytes (white blood cells [WBCs]) [18,19,20]. CSF is produced by the choroidal plexus of the ventricular system and ependymal brain cells from blood [18,20,21].

In tumor patients with CNS involvement, CSF contains various markers associated with disease progression and responses to therapy [2,3,4,13,14,15,16,17,22,23,24,25,26,27,28,29,30]. Among others, circulating tumor cells (CTCs) are direct seeds of metastasis and, therefore, their diagnostic significance encourages high attention of researchers and clinicians. Furthermore, multiple recent reports suggested that detection of tumor-derived markers such as exosomes, circulating tumor DNA (ctDNA), micro-RNA (miRNA) and proteins is relevant to LM, BM and BT. The diagnostic significance of these markers seems especially important for BT because some BTs are not metastatic and do not typically shed CTCs but may release tumor-derived markers in CSF. CTC aggregates (so-called clusters or emboli) in CSF may also have diagnostic value. This speculation is based on: (1) finding CTC emboli in CSF samples of patients with lung cancer and LM [30]; (2) detection of CTC clusters in blood of patients with BT (e.g., glioblastoma) assuming their leaving CNS through the compromised blood-brain barrier (BBB) [31]; and (3) experimental and clinical evidences that multicellular CTC aggregates in peripheral blood represent the aggressive cell subset responsible for initiating and promoting metastasis [31,32,33,34,35,36,37,38,39,40].

Based on the physiology of CNS and mechanisms of tumor development (e.g., compromising BBB to penetrate tumor cells [41]), CTCs, their aggregates and other tumor-derived markers may invade CSF through different mechanisms that include (1) crossing the compromised BBB by blood and lymphatic CTCs and/or (2) shedding tumor cells by existing BM and BT. The latter mechanism provides a solid basis for using CSF tumor markers to diagnose progression of BM and BT, and to estimate responses to therapy. The first mechanism likely works for LM and BM and suggests the origin of CSF tumor biomarkers from blood or/and lymph and their possible entry to CSF before colonization of brain tissue and meninges.

Thus, testing CSF might predict deadly LM, BM and BT; and advanced methods to assess CSF tumor markers in CSF are urgently needed to prolong life of patients suffering from CNS tumor lesions.

## 2. In Vitro Detection of CSF Tumor Markers

The gold standard for routine clinical examination of CSF is cytology after lumbar puncture [9,10,11,24,42,43]. The detection approach is based on cytomorphology of tumor cells after staining samples with Wright-Giemsa or Papanicolaou dyes. However, the sensitivity of CSF cytology is estimated as low as 50% [9]. Furthermore, cytology is a relatively subjective method since its results depend on the ability of a laboratory technician to correctly identify types of cells, for example, to distinguish tumor cells from normal leukocytes [24,25,26]. This may lead to delaying of therapeutic interventions until other diagnostic criteria (e.g., abnormal magnetic resonance imaging [MRI]) and/or strong clinical symptoms emerge. As a result, involvement of CNS in some patients is found at autopsy only.

**Figure 1:**
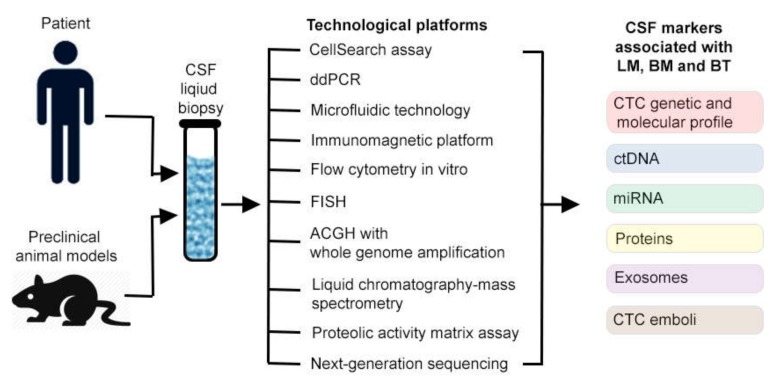
Cerebrospinal fluid (CSF) liquid biopsy detection of tumor markers in vitro.

The limitations of cytology and deadly nature of LM, BM and BT encouraged researchers and clinicians to develop more sensitive and accurate markers using modern technologies. During the past decade, substantial efforts have been made to assess CSF samples using new concept of liquid biopsy (Figure 1) [2,15,16,17,23,26,27,28,29,30,44,45,46,47,48].

### CSF Liquid Biopsy

Several years ago, Patel et al. showed that FDA-approved CellSearch method can be used to identify CTCs in 7.5 mL CSF samples of breast cancer patients [22]. Compared to traditional cytology, the CellSearch assay has been demonstrated significantly higher number of CTCs [22,28,30,49,50]. Despite promise, this technological platform is limited in detection of only a few tumor markers, typically EpCam, for patients with epithelial cancers (e.g., breast cancer) and CD 146 and HMW-MMA for patients with melanoma [21,49]. Thus, CellSearch obviously cannot identify a highly heterogeneous population of CTCs and not suitable for diagnosis of many tumors such as glioblastoma. These limitations somewhat reduced enthusiasm to recommend this method in routine clinical practice.

Using real-time polymerase chain reaction (RT-PCR) for examination of patients with BM and LM has demonstrated higher sensitivity than conventional cytology [51]. However, relatively high rate of false-negatives during RT-PCR analysis make it a suboptimal method for CSF testing [9]. To solve this problem, cancer researchers and clinical oncologists recently explored the use of high-sensitive droplet-digital PCR (ddPCR) [52,53,54,55,56]. It was shown that ddPCR provides accurate and reliable CSF analysis. It can work with poor DNA quality and measure multiple parameters including absolute allele quantification, rare mutation, copy number variations, DNA methylation and gene rearrangements [52]. In a few clinical studies, ddPCR of CSF was able to detect ctDNA in patients with melanoma and CNS metastasis; and the obtained results were strongly correlated with cytology results and detection of abnormalities in MRI [52,56]. It is interesting that some patients with high level of ctDNA showed negative cytological results [56]. The small volume of CSF fluid required for testing ctDNA is definitely an additional advantage but high level of false results is a challenge. Overall, to date, it is too early to make conclusions on diagnostic value of ctDNA.

Another promising emerging data of CSF liquid biopsy have been obtained using immunofluorescence in situ hybridization (FISH) technology [24,57,58,59,60]. The published results hold promise to provide more accurate diagnosis of CSF CTCs than cytology. The main advantage of FISH is phenotypic and karyotypic identification and characterization of the highly heterogeneous CTCs, which can be assessed by both chromosome ploidy and the expression of various tumor markers [57]. However, FISH is not currently standardized for liquid biopsy and requires future development and research to clarify whether or not this method is reliable for identification of CTCs.

Integration of array comparative genomic hybridization (ACGH) analysis and whole genome amplification provided achieving the genomic characterization of rare CSF CTCs [61,62]. The clonal similarity between CSF CTCs and primary tumor genomic profiles with more copy number alterations in CTCs was demonstrated using samples of CSF and primary tumor from breast cancer patients with LM [62].

Analysis of CSF samples with conventional flow cytometry in vitro has been reported to diagnose CTCs in CSF [63,64,65]. Flow cytometry immunophenotypic testing of bulk breast cancer receptors, cancer stem cell markers and various WBC subpopulations looks interesting and suggests interplay of CSF and lymph fluid during CTC migration [63]. However, well-known limitation of flow cytometry to detect rare events might reduce enthusiasm for its use of assessment of CTCs which is supposed to be rare (up to 1-5 CTCs per sample) at early stage of CNS involvement.

In the past few years, the clinical potential of some other technological platforms including microfluidic technology, immunomagnetic platform, high performance liquid chromatography-mass spectrometry, next generation sequencing (NGS) and proteolytic activity matrix assay (PrAMA) has also been demonstrated [25,50,55,66,67,68,69]. Despite interest and promises, the singularity of these reports does not allow yet making conclusions on suitability of these methods to improve prognosis in patients.Overall, despite CSF liquid biopsy is expected to yield clinically significant biomarkers and assays, the main drawback to all aforementioned approaches in vitro is that their sensitivity is substantially limited by the volume of the sample [70,71]. Typically, up to 10 mL of CSF is used for examination, which is estimated to be less than 6–7% of the total 130–150 mL volume of human CSF. It means that in vitro testing misses up to 93–94% of CTCs [71]. A simple recalculation of the results in vitro, which detected minimum 1–2 CTCs per CSF patient sample (5–10 mL) with the existing LM and BM [21,49], shows that the real number of CTCs at the time of diagnosis was more than 15–20 cells in the total CSF volume. Serial analysis of multiple samples from repeated punctures increases sensitivity [28]. However, repeated punctures are a challenge because it can be performed over several days and may lead to delaying of therapies. In addition, the existing methods in vitro are burdened with: (1) low throughput, which may require many hours (if not, days) to assess a typical CSF sample and (2) multiple time-consuming sample-processing steps including staining, immunomagnetic capture, isolation and washing, which result in loss of many CTCs [21,23,30,49,51]. As a result, CTCs in small quantities may escape detection, which also contributes to late diagnosis and poor outcomes.

Based on this, liquid biopsy in vitro can provide advanced molecular and genetic analysis of tumor associated markers in CSF but it cannot detect rare CTCs at early stage of LM and BM and possibly, before LM and BM initiation (Table 1). The rarity of CSF CTCs definitely demands a new strategy. An attractive solution to these problems is to monitor almost entire CSF volume in vivo (Table 1).

## 3. In Vivo Diagnosis of CSF

Despite significant progress in neuroimaging in vivo (e.g., MRI, computed tomography [CT], radiography) [9,11,50,64,72,73,74], existing diagnosis, even advanced multi-modal imaging is not sufficient to make judgments about early LM and BM. The low spatial and temporal resolution of CT and MRI allows identification of only macroscopic changes in the CNS (e.g., metastases ≥10 mm by CT). Therefore, the diagnosis is typically based on the indirect signs of LM including pathological meningeal contrast enhancement at the MRI examination, which are often equivocal. In addition, a recent study has found that immunotherapy might be a source of MRI false positivity (‘pseudomeningeosis’) [73]. New generations of MRI, such as phase-contrast MRI, enable quantitative measurements of CSF flow but not suitable for detection of relatively fast moving single CTCs and particles due to slow time response [75]. The same limitation applies to intravital fluorescence microscopy which has been used for imaging CSF plasma (so-called, cisternography) but not single cells in CSF [76]. Furthermore, the translation of fluorescent neuroimaging to humans in vivo is problematic due to (1) cytotoxicity of fluorophores, (2) undesirable immune responses to tags and (3) assessing only superficial fluid flows due to strong influence of autofluorescent and scattering background.

### Photoacoustic Flow Cytometry In Vivo

**Figure 2:**
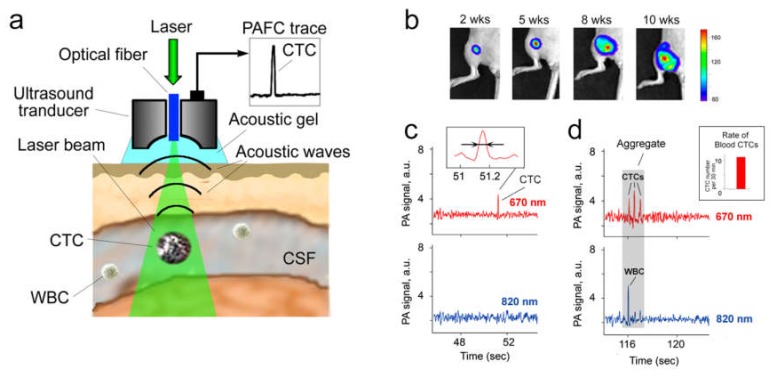
Assessment of circulating tumor markers in CSF in vivo with multicolor PAFC. (**a**) Principle of diagnosis with PAFC. (**b**) Intravital luminescence imaging of metastatic breast cancer progression in orthotopic xenograft mouse model after inoculation of human MDA-MB-231-luc2-GFP cells. (**c**) Two-color PAFC of the spontaneous CSF CTCs in vivo; inset: the photoacoustic signal width (indicated by arrows), which is associated with a single circulating tumor cell (CTC). (**d**) PAFC of circulating CTC-containing embolus in tumor-bearing mice; gray rectangle: aggregate of CSF-CTCs and leukocyte (WBC); insert: the blood CTC rate at the time of CSF monitoring.

The most promising method for detecting CTCs in CSF is photoacoustic (PA) flow cytometry (PAFC), which compared to other in vivo diagnostic techniques, demonstrated ultra-sensitive molecular detection and counting of single cells in different body fluids (e.g., blood, lymph and CSF) [40,70,77,78,79,80,81,82,83]. The principle of multicolor PAFC is based on noninvasive (i.e., through intact skin) irradiation of the selected fluid with short laser pulses at different wavelengths followed by the detection of laser-induced acoustic waves (referred to as PA signals) using an ultrasound transducer placed on the skin (Figure 2a). PA methods provide higher sensitivity and resolution in deeper tissues (up to 2–3 cm, with potential up to 5–7 cm [70,84]) than other optical modalities. These benefits make possible detection of CTCs in CSF through the atlanto-occipital membrane. In PAFC, this allows distinguishing signals from single fast-moving particles (e.g., CTCs, exosomes, and emboli) at laser energies within the safety standards for humans [70,77,81,83,85,86]. In regards of CSF detection, PAFC has advantages compared to other in vivo methods. Specifically, the colorlessness and optical transparency of CSF, commonly accepted as a diagnostic limitation, provides low absorbance and, therefore, extremely low PA background signal, which significantly improves distinguishing stronly light absorbing objects [71]. It means that CTCs, exosomes or emboli with strong absorbing molecules (e.g., natural melanin or nanoparticles) are predominated over the absorption of CSF by a few orders of magnitude, especially in the near-infrared window of transparency for biotissues (“first window”: 700–1100 nm). Based on this, some strong absorbing cells such as melanoma CTCs with natural intracellular high absorbing melanin as intrinsic non-toxic PA contrast agents, can be easily detected by PAFC in label-free mode. To detect low absorbing tumor-related CSF markers (e.g., breast cancer CTCs), they should be labeled by exogenous PA contrast agents conjugated with ligands (e.g., antibodies, peptides, or folic acid) against specific surface receptor(s). The key requirements for in vivo use of contrast agents include low toxicity and high PA contrast. Some of the best candidates are gold and magnetic nanoparticles [77,87,88].

The first successful demonstration of PAFC’s capability to diagnose CSF tumor markers was reported using preclinical models of breast metastatic cancer (Figure 2b–d) [71]. It was shown that PAFC was able to detect CSF CTCs with 10–20 times higher sensitivity compared to in vitro methods. The most important finding is that some tumor-bearing mice without histologically detectable BM exhibited rare CSF CTCs (e.g., 1–3 signals every 40–60 min). The presence of blood CTCs in these mice suggests the possible origin of CSF CTCs to be from blood CTCs and indicates the potential of CSF CTCs as a predictive biomarker of BM. The obtained experimental evidence is in line with the aforementioned suggestion that blood and lymphatic CTCs might pass the compromised BBB and enter brain tissue, meninges and CSF to form BM and LM. This may serve as a scientific foundation for prognosis and prediction of LM and BM in patients.

Another interesting finding is the existence of CTC-containing emboli in CSF in vivo (Figure 2d). Identification of embolus is based on the width and shape of PA signal, assuming that embolus’ multicellular structure produces a relatively wider PA signal containing a set of narrower peaks.

Overall, the success of preclinical studies together with the simplicity and safety of PAFC give confidence to rapidly translate this method into clinical practice. PAFC diagnosis of CSF in human subarachnoid space and spinal canal at a depth of 1–3 cm seems possible and was supported by the reports on high sensitivity and resolution of PA methods in deeper tissues. Recently, the clinical relevance of PAFC was successfully demonstrated in clinical trials with melanoma patients by detecting blood CTCs in 1–2 mm hand vessels at depth of 1-3 mm with a detection limit of 1 CTC/1000 mL (i.e.,10^3^ –fold increased sensitivity compared to existing CTC assays) [40].

## 4. Future Directions

To date, crucial steps in increasing the survival of patients with LM and BM are (1) early diagnosis; (2) initiating preventive therapy such as targeted therapy of single CSF CTCs and their emboli and (3) assessing therapeutic efficacy in order to optimize an individual course of therapy.

### 4.1. Advance Diagnosis

Although many promising technologies to detect various CSF tumor markers during liquid biopsy have been reported, there is no standardized and validated assay that is currently ready to introduce for daily clinical practice as an advanced alternative or supplement of conventional cytology.

Novel approaches integrating unprecedented high sensitivity of in vivo flow cytometry and comprehensive molecular and genetic characterization of tumor markers in CSF in vitro are highly desired for clinical needs.

In addition, one of the possible future alternatives is CSF diagnosis in vivo using updated GILUPI CellCollector. This method was introduced in 2016 for EpCam-based detection of CTCs in blood by introduction of EpCAM-coated wire into a vein of the patient [89]. However, the invasive nature of the method and possibility of missing CTCs, which transit outside the wire, somewhat reduce enthusiasm of using GILUPI device for CSF assessment.

The new looks are also suggesting continuous cell exchange between CSF, blood, lymph and brain tissue [90,91,92] that should be considered at the diagnosis. The prognostic value of CTCs, if they are simultaneously tested in blood, lymph and CSF, would provide a new, highly sensitive and accurate prognostic biomarker of metastasis progression and therapy efficacy.

### 4.2. Therapeutic Perspectives

Minimal treatment options in current management of LM and BM lead to poor prognosis for patients due to low efficacy, late therapy initiation, use of common (i.e., not-personalized) therapeutic schematics and high toxicity. From this, one of the top future priorities is development of novel targeted and immune therapies. The molecular-targeted nanotechnology platform is highly promising for targeted drug delivery. For this purpose, nanoparticles should have high sensitivity, specificity and selectivity as well as safety, multifunctionality, multimodality, ability to penetrate BBB and high efficiency of drug delivery to tumor. Among existing nanoparticle-based drug cargoes, the most promising candidates include low toxic individual nanoparticles, high-contrast spasers, liposomes, polymer micelles, lipid micelles packaged with semiconducting polymer dots as simultaneous MRI and PA imaging and photodynamic and photothermal dual-modal therapeutic agents, layer by layer based composite structures (core-shells) and microcapsules (shells) and biocompatible natural magnetic nanoparticles [87,88,93,94,95,96,97,98]. The targeting could be achieved by surface modification using targeted molecules specific to CTCs, exosomes and emboli [88,99]. For example, a single injection of core-shells in CSF has shown the effectiveness of their use for the long-term delivery of painkillers in the treatment of persistent pain [100]. Potentially, these drug delivery systems may be effective for treating CTCs in CSF.

There is a high therapeutic potential of modern technologies for creating synthetic truncated antibodies [101] and scaffolds [102]. The revolutionary progress in genetic and protein engineering methods make it possible to directionally modify the molecular size, affinity, specificity and immunogenicity of an antibody, their derivatives and analogues, oriented to the use in the diagnosis and targeted therapy of cancer. Today, rational design and molecular engineering allow modelling of the compounds with preprogrammed properties and to create biotechnological producers of therapeutic medicines [102,103,104,105,106]. A promising direction is conjugation of these unique theranostic agents with nanoparticles. The advantages of using nanoparticles in these conjugates include developed surface of nanoparticles, which can be decorated with biocompatible functional moieties for targeted delivery; and diagnosis that guides and monitors effects of the nanoparticle-assisted therapy [107,108,109,110]. Recently, the design of a hybrid nanocomplex based on an upconversion nanoparticle (UCNP) was reported [111]. Owing to their unique photophysical properties, UCNPs are high-potential platform for theranostics complexes. Conversion of near-infrared light, which can deeply penetrate in biological tissue, to the higher photon energy visible, ultra-violet and near-infrared light is among UCNP’s most useful properties. Two toxic agents––beta-emitting radionuclide yttrium-90 and a highly efficient targeted toxin DARPin-exotoxin A from Pseudomonas aeruginosa––were coupled to UCNP core to exert toxicity to cancer cells. As a result, on the one hand, the photophysical properties of hybrid nanocomplex enable background-free imaging of its distribution in cells and animals. On the other hand, specific delivery of UCNP complexes to cancer cells results in combined therapy by two toxic agents with markedly increased synergetic effect [111]. The design of the hybrid multifunctional nanoheterocomplex proves the principle “when the whole is greater than the sum of the parts.”

The novel targeted CSF therapy may also use the advanced design of heterostructures based on the barnase:barstar pair [112]. The ribonuclease barnase and its inhibitor, barstar, are small stable proteins. They form extremely tight complex with a K_d_~10^−14^ M. The strategy is applicable to any proteins or nanoparticles that can be functionally attached to the barstar and barnase, especially for production of heterooligomeric constructs because the extremely specific barnase barstar interaction eliminates reliably the mispairing problems. This universal platform is a promising alternative to commonly used chemical conjugation techniques in nanobiotechnology, theranostics and clinical applications. It provides a straightforward technology to design wide range of multifunctional nanoheterostructures for the highly efficient delivery of active agents to tumor cells for theranostics [112,113,114,115,116,117].

A very exciting future direction is the possibility of integration of in vivo molecular diagnosis, targeted therapy and estimation of therapeutic efficacy in one technological platform of PAFC [40,79,118]. PAFC’s capability to identify a single high-absorbing CTC and immediately “kill” it through photothermal-indiced nanobubbles with photomechanical action on CTC membranes and vital intracellular structures was demonstrated for blood CTCs in experiments and, recently, in clinical research in blood circulation [40]. Furthermore, the following disappearance of the CTC-associated PA signals might serve as the criterion of effective therapy. These data bring hope that earliest rare CTCs might be identified and “killed” directly in CSF before colonization of brain tissue and formation of BM and LM.

It is expected that technological innovations and ongoing clinical trials would contribute to the finding of novel approaches to provide advances in BM and LM theranostics at the earliest possible stages before development of overt deadly lesions, to select patients with high risk of BM and LM for personalized therapy, to identify early disease progression and thereby improve survival rates of cancer patients.

## Figures and Tables

**Table 1:** New and emerging technologies for detection of tumor biomarkers in CSF.

Detection Method	Biomarker	Disease	Approach		Significant Advantages	Main Limitations	Application		Refs
			In Vitro	In Vivo			Research	Clinical	
CellSearch	CTCs, Cell emboli	BM, LM	+		FDA-approved technology, Higher sensitivity than cytology	Small sample volume, Processing delay, Limited number of detected markers	+	+	[22,28,30,49,50]
Microfluidic technologies	CTCs	BM, BT	+		Single CTC capture in sub-nanoliter trap, Relatively quick (~1 h) analysis	Early stage of research using cell lines	+		[69]
Immuno-magnetic platform	CTCs	LM	+		Capable to detect and separate rare cells	Low sensitivity due to small sample volume, Limited number of detected markers		+	[25]
FC in vitro	CTCs	LM	+		Standar-dized technology, Higher sensitivity than cytology	Impossibility to detect rare cells	+	+	[63,64,65]
ddPCR	ctDNA, miRNA, CTCs	BT, BM, LM	+		High specificity	False-positivity, Not standardized	+	+	[52,53,54,55,56]
FISH	CTCs	LM, BT	+		Analysis poor DNA, Relatively high resolution	Early stage of research, Not standardized	+	+	[24,57,58,59,60]
ACGH	CTCs		+		Whole genome sequencing, High resolution compared to conventional CGH	Inability to detect aberrations that do not result in copy number changes		+	[61,62]
NGS	ctDNA, miRNA	LM	+		High-throughput whole genome sequencing, High specificity	High price, Complex data analysis		+	[50,56]
PrAMA	Proteases	LM	+		Detection of protease activity as indicator of BBB degradation	Early stage of research		+	[66]
PAFCin vivo	CTCs, Cell emboli	BM			Extremely high sensitivity, Theranostic capability	Detection of surface CTC receptors	+		[71]

CTCs—circulating tumor cells, LM—leptomeningeal metastasis; BM—brain metastasis; BT—brain tumor; FC—flow cytometry; ddRCP—droplet-digital polymerase chain reaction; ctDNA—cell free DNA; miRNA—microRNA; FISH—immunofluorescence in situ hybridization; ACGH—array comparative genomic hybridization; CGH—comparative genomic hybridization; NGS—next generation sequencing; PrAMA—proteolytic activity matrix assay; PAFC—photoacoustic flow cytometry.
